# Combretastatins: An Overview of Structure, Probable Mechanisms of Action and Potential Applications

**DOI:** 10.3390/molecules25112560

**Published:** 2020-05-31

**Authors:** Gökçe Şeker Karatoprak, Esra Küpeli Akkol, Yasin Genç, Hilal Bardakcı, Çiğdem Yücel, Eduardo Sobarzo-Sánchez

**Affiliations:** 1 Department of Pharmacognosy, Faculty of Pharmacy, Erciyes University, 38039 Kayseri, Turkey; gskaratoprak@erciyes.edu.tr; 2 Department of Pharmacognosy Faculty of Pharmacy, Gazi University, 06330 Ankara, Turkey; 3 Department of Pharmacognosy, Faculty of Pharmacy, Hacettepe University, 06100 Sıhhiye, Ankara, Turkey; ygncyasin@gmail.com; 4 Department of Pharmacognosy, Faculty of Pharmacy, Acibadem Mehmet Ali Aydınlar University, 34752 Istanbul, Turkey; hilal.bardakci@acibadem.edu.tr; 5 Department of Pharmaceutical Technology, Faculty of Pharmacy, Erciyes University, 38039 Kayseri, Turkey; cyucel@erciyes.edu.tr; 6 Instituto de Investigación e Innovación en Salud, Facultad de Ciencias de la Salud, Universidad Central de Chile, Santiago 8330507, Chile; eduardo.sobarzo@ucentral.cl; 7 Department of Organic Chemistry, Faculty of Pharmacy, University of Santiago de Compostela, 15782 Santiago de Compostela, Spain

**Keywords:** combretaceae, combretastatins, drug discovery, natural compound, nanoformulation, structure-activity relationships, tubulin inhibitors

## Abstract

Combretastatins are a class of closely related stilbenes (combretastatins A), dihydrostilbenes (combretastatins B), phenanthrenes (combretastatins C) and macrocyclic lactones (combretastatins D) found in the bark of *Combretum caffrum* (Eckl. & Zeyh.) Kuntze, commonly known as the South African bush willow. Some of the compounds in this series have been shown to be among the most potent antitubulin agents known. Due to their structural simplicity many analogs have also been synthesized. Combretastatin A4 phosphate is the most frequently tested compounds in preclinical and clinical trials. It is a water-soluble prodrug that the body can rapidly metabolize to combretastatin A4, which exhibits anti-tumor properties. In addition, in vitro and in vivo studies on combretastatins have determined that these compounds also have antioxidant, anti-inflammatory and antimicrobial effects. Nano-based formulations of natural or synthetic active agents such as combretastatin A4 phosphate exhibit several clear advantages, including improved low water solubility, prolonged circulation, drug targeting properties, enhanced efficiency, as well as fewer side effects. In this review, a synopsis of the recent literature exploring the combretastatins, their potential effects and nanoformulations as lead compounds in clinical applications is provided.

## 1. Introduction

Throughout the ages humans have utilized Nature, especially plants, to meet their basic to complex needs such the production of foodstuffs, shelter, clothing, means of transportation, fertilizers, flavors, fragrances and medicines as well. The World Health Organization has revealed that approximately 80% of the world’s people count on medicinal plants in order to maintain their health or for treatment purposes. Newman et al. reviewed natural products as sources of new medicines between 1981 and 2002. This study showed that 67% of 877 mentioned small molecule new chemical entities were synthetic, 16.4% were synthetic molecules with natural product skeletons and 12% are literally modeled on a natural product inhibitor of the target or mimic the endogenous substrate of the active site [1,2].

Cancer is a malignant disease characterized by uncontrolled abnormal cell division. Uncontrolled proliferating cells are then able to penetrate other tissues, thus resulting in metastasis. The demand for effective and curative cancer chemotherapeutic agents for such neoplastic diseases is crucial. Evaluation of natural products as anticancer agents was recognized in the 1950s by the U.S. National Cancer Institute (NCI), and the NCI has since made major contributions to the discovery of new naturally occurring anticancer agents. Up to date, it is known that more than 60% of the molecules used in cancer therapy originate from plants or other natural sources [3].

As a part of NCI’s anticancer agent discovery research, among the *Combretum* species (Combretaceae), both *C. molle* and *C. caffrum* were found to be significantly active against murine P-388 lymphocytic leukemia [4]. The promising results of the NCI pioneered the isolation of bioactive constituents from members of the *Combretum* genus, especially *C. caffrum*. Pettit et al. conducted further phytochemical and biological activity studies on root barks of *C. caffrum* in collaboration with the NCI Natural Products Branch and succeeded in the isolation of a compound named combretastatin [5]. After the discovery that combretastatin was inactive, studies were focused on uncovering bioactive combretastatin derivatives (combretastatin A-1, B-1 and the most potent A-4) [4,6,7].

Combretastatins are a series of closely related stilbenes (combretastatins A), dihydrostilbenes (combretastatins B), phenanthrenes (combretastatins C) and macrocyclic lactones (combretastatins D). Despite their similar name, combretastatins are not related to the statins, a type of cholesterol-reducing drugs. Common structure of combretastatins have three common characteristics: a trimethoxy “A”-ring, a “B”-ring comprising substituents frequently at C3′ and C4′, and generally an ethene bridge amongst the two rings providing the rigidity of the structure. If there is an ethene bridge between the A and B ring in combretastatin structure are stilbenoids, metabolites without a double bond on the bridge are dihydrostilbenoids [8].

A phenanthroquinone, a class of compounds rarely found as biosynthetic products, has been isolated from the same extract and called combretastatin C-1. Combretastatins D-1, D-2, D-3 and D-4 belong to a different type of compounds isolated from both *C. caffrum* and *Getonia floribunda* Roxb. Lam., whose prominent structural feature is a macrocyclic lactone [9,10,11,12,13].

What makes this class of compounds (primarily the A series) rather more interesting than other secondary metabolites is their significant antimitotic and antiproliferative activity. The activity of combrestatins is closely related to their stereoisomeric configurations. Both natural and synthetic derivatives with diverse configurations and functional groups have been a main subject of cancer chemotherapy clinical trials in recent years [14,15]. The aim of this study is to review the occurrence, bioactivities, structural varieties and structure activity relationship studies of combretastatins.

## 2. Occurrence of Combrestastatins

**Figure 1:**
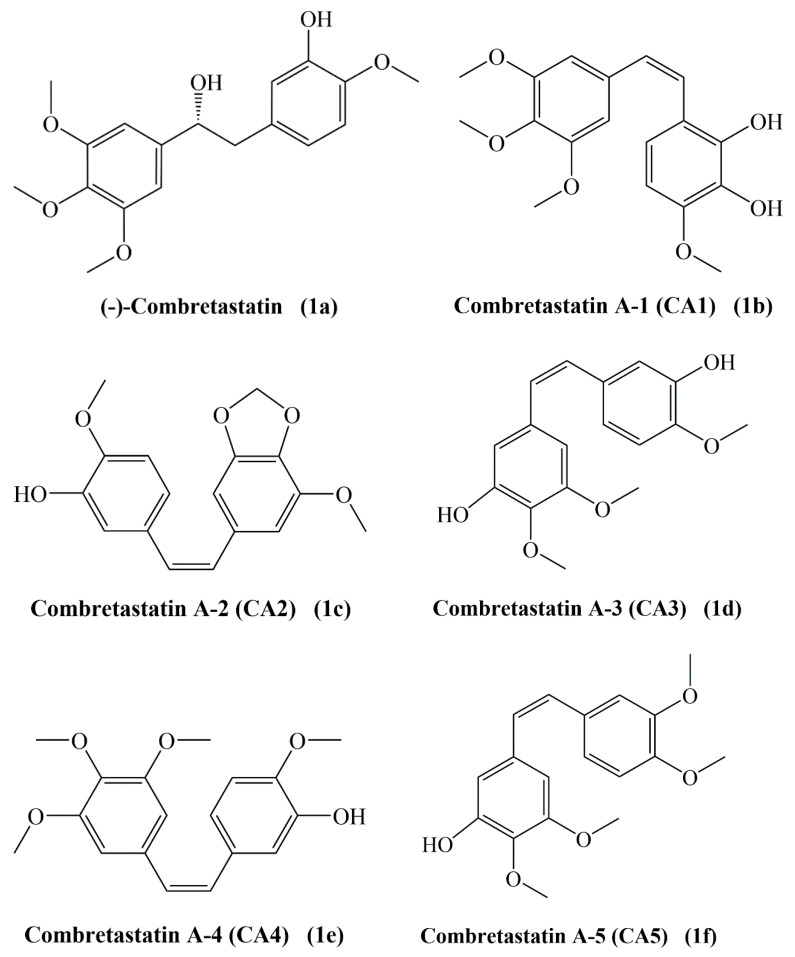

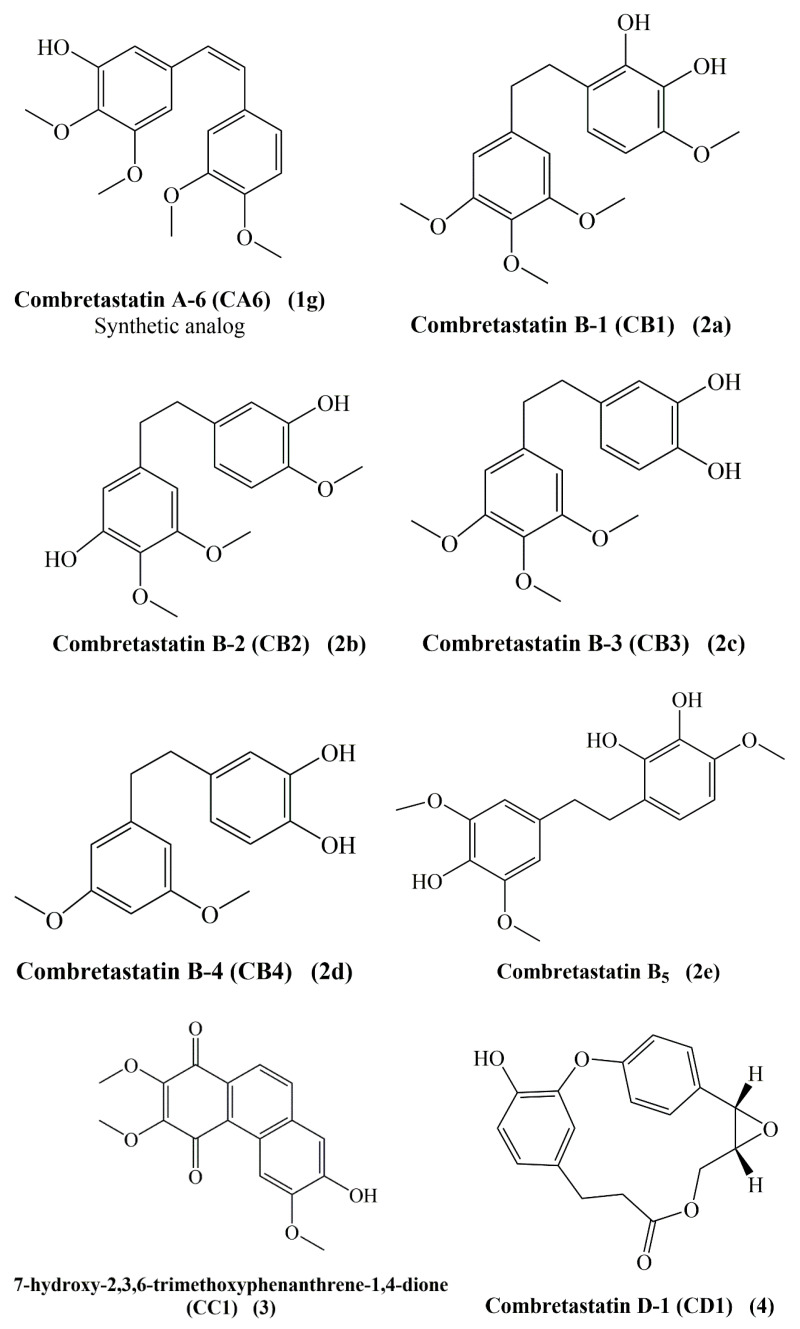

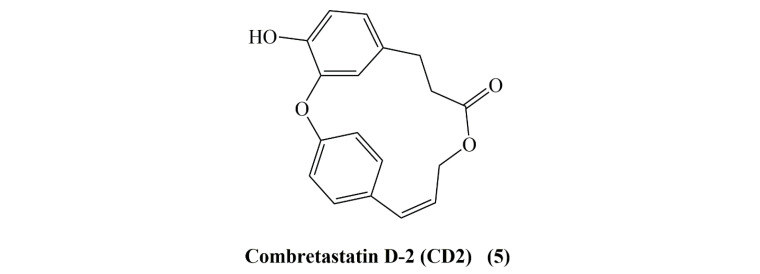
Structures of combretastatins.

Combretastatins are a series of bioactive stilbenes (combretastatins A series), dihydrostilbenes (combretastatins B series), phenanthrenes (combretastatins C series) and macrocyclic lactones (combretastatins D series). Representative members of each of these groups are shown in Figure 1. Combretastatins display three main structural features: a trimethoxy “A”-ring, a “B”-ring containing substituents often located at C3′ and C4′, and an ethene bridge (stilbenoids) between the two rings which contributes to the necessary structural rigidity. Both phenyl rings are tilted at 50−60° with respect to each other and are linked by a two-carbon bridge. Combretastatins with such an ethene bridge are called stilbenoids; molecules with a non-ethene bridge are called dihydrostilbenoids [8]. There are six members in combretastatin A family (CA1 to CA6) (**1a**–**1f**) with *cis*-(CA1-CA5) and *trans*-stilbene (CA6) (**1g**) moieties. The B series contains four types CB1 (**2a**) to CB4 (**2d**) [13]. A phenanthroquinone, a class of compounds rarely found as biosynthetic products, has been isolated from the same extract and called combretastatin C-1 (**3**). Combretastatins D-1 (**4**), D-2 (**5**), D-3 and D-4 belong to a different type of compounds isolated from both *C. caffrum* and *G. floribunda*, whose prominent structural feature is a macrocyclic lactone [10,11,12,13,16]. A great number of combretastatins were primarily isolated from the barks of the South African willow tree *Combretum caffrum* [4,5,6,11,16,17,18]. The interest in combretastatins (mainly A series) is due to their potent antitumor properties by inhibiting tubulin polymerization and tumoral vasculature formation disruption (vascular targeting agents). Among them combretastatin A-4 (CA-4, **1e**) was found to be the most potent antitumor agent against NCI-60 human tumor cell lines, followed by combretastatin A-1 and A-2 [19].

**Figure 2:**
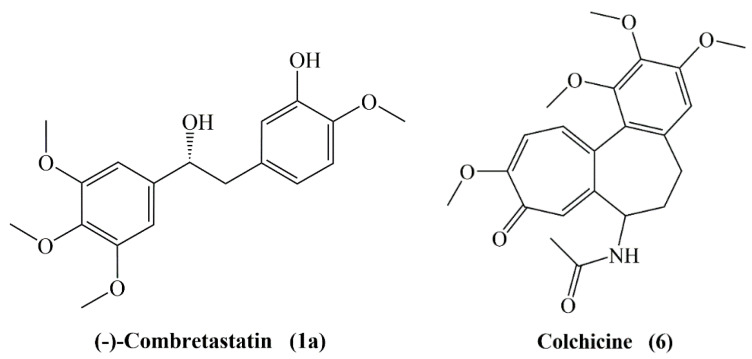
Structural similarity between combretastatin (**1a**) and colchicine (**6**)**.**

Combretastatins have matching molecular structures with colchicine (**6**), as both contain a trimethoxyphenyl ring and the aromatic tropone ring of colchicine is related to the isovanillinyl group of combretastatins (Figure 2). The most promising antimitotic combretastatin is the A-4 type. It readily binds to the tubulin at the colchicine site. Structure activity relationship (SAR) studies revealed that the *cis* configuration of the stilbene moiety and a double bond with two rings are important features for inhibition of tubulin polymerization. The olefinic bond allows the placement or aromatic rings in an appropriate way and imparts the molecule the required flexibility to achieve the right dihedral angle to maximize its interaction with the binding site. Indeed, a *Z* configuration of the double bond is crucial for the activity. Studies showed significant decrease in the activity was seen in *E*-stilbenes when compared with the *Z*-stilbenes [20,21,22].

**Figure 3:**
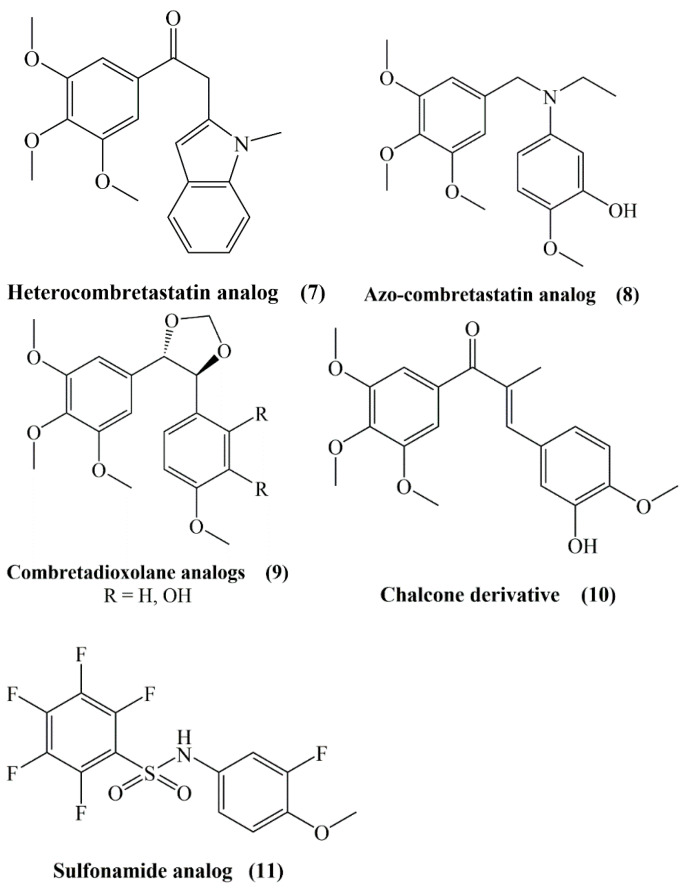
Structures of heterocombretastatin, azo-combretastatin, combretadioxolane analogs, chalcone derivatives and sulfonamide analogs.

Poor water solubility of CA-4 led to the synthesis of several products preserving the A and B aryl rings and ethylene bridge with functional group modifications such as heterocombretastatins (**7**), azo-combretastatins (**8**), combretadioxolane analogs (**9**), chalcone derivatives (**10**) and sulfonamide analogs (**11**) (Figure 3) [13].

Benzophenone derivatives of CA-4 showed less cytotoxicity than CA-4. SAR studies revealed that the addition of an amino group at the *ortho* position of the B ring increased the cytotoxicity, but a hydroxyl and N-methylated groups decreased the activity [20]. With its remarkable vascular targeting activity the simple disodium phosphate monoester (4-*O*-phosphate derivative (CA-4P), developed by OXiGENE (Waltham, MA, USA) to treat anaplastic thyroid cancer in combination with other anticancer drugs and also for myopic macular degeneration) with improved solubility is the most studied one. The short circulation half-life of CA-4P has prompted the synthesis of longer-lived forms [23]. The CA-4 dialkylphosphate triesters, anionic alkyl and aryl phosphate diesters, phosphate diester, the phosphate-linked CA-4 dimer and the ether-linked alkyl sulfonate salt were synthesized and tested for their cytotoxicity and vascular activity, although only the phosphate diesters were found to have similar to higher activity compared to CA-4P with lower cytotoxicity [23]. Most modifications of the B ring displayed lower bioactivity, but nonetheless removal of 3′-OH function exhibited the similar potential with CA-4 in tubulin polymerization and colchicine binding. Correspondingly, replacement of the 3’-OH with an amino group resulted in equivalent bioactivity with CA-4 and developed water solubility as well [24].

Furthermore, Cushmann et al. synthesized *cis*- and *trans*-stilbenes, dihydrostilbenes and N-arylbenzylamines and evaluated against cell lines in the context of SAR studies. Similarly with the previous studies, *cis*-stilbenes were more potent than the other groups of compounds, against A-549 lung carcinoma, MCF-7 breast carcinoma, HT-29 colon adenocarcinoma, SKMEL-5 melanoma, and MLM melanoma cell lines, though being a 20 times less potent than CA-4. The presence of the 4-methoxy function was found to be the crucial for the activity [25]. As a summary, the presence of 3,4,5-trimethoxy groups at ring-A, 4′-methoxy group at ring-B and *cis*-configuration of the olefin are the crucial elements for excellent tubulin assembly inhibition [21,24,26].

## 3. Preclinical Studies on Combretastatins

### 3.1. Antioxidant Activity

Resveratrol, which is known for its strong antioxidant activity, has a *trans*-stilbene structure and its methylated analog is also known to have strong protective effects against hydroxyl radical and radical-based DNA damage [27]. Considering that synthetic stilbenes exhibit similar activities, the antioxidant activities of combretastatins and synthesized analogs, which have a stilbene structure, were also evaluated. In the study, CA-4 (**1e**) and polymethylenedioxy analogs of combretastatin A2 (**1c**) were synthesized and cytotoxic activities in different cancer cell lines and antioxidant activities were evaluated. CA-4 has been reported to exhibit strong antioxidant effects compared to other analogues in ABTS and DPPH tests. It was found that both the *cis* and *trans* isomers of CA-4 had DPPH radical scavenging IC_50_ values of 4.65 mg/mL and 5.71 mg/mL, respectively, and the IC_50_ value of ascorbic acid used as reference was 8.43 mg/mL [27].

**Figure 4:**
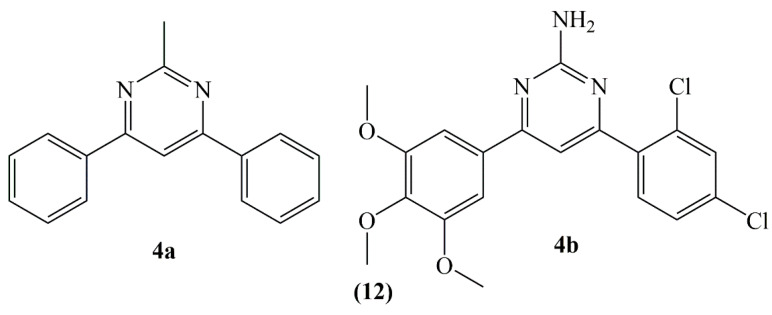
Structure of diphenyl pyrimidine analogues **4a** and **4b**.

In a study pyrimidine ring was added as a 3-carbon binder to sustain the *cis*-locked conformation of CA-4. Thus, diphenylpyrimidine analogues 4a and 4b (**12**) were synthesized by binding different substituents to ring A and ring B (Figure 4). Compound **4a** which is the least substituted and highly substituted compound **4b** (trimethoxy substituents in ring A), revealed strong activity towards A549 (lung) and MCF7 (breast) cancer lines and hence they were also investigated for their antioxidant activities in these cell lines. The effects of compounds **4a** and **4b** on superoxide dismutase (SOD), catalase (CAT) and glutathione reductase (GR) enzymes which were involved in free radical detoxication were measured. It has been reported that CAT and GR activities decreased markedly in the groups in which compounds **4a** and **4b** were administered, while there was no meaningful difference in SOD activity and direct correlation between glutathione amounts and proliferation of cells. It is also stated that the compounds do not have DPPH radical scavenging properties. According to the results of the study, it has been showed that the compounds can disrupt the cellular antioxidant defense system and trigger the apoptotic pathway by causing increased ROS in the cell [28].

Sadanand et al., synthetized 3,5-diaryl-1-carbothioamide-pyrazoline, N1-phenylsulfonyl pyrazoline and pyrimidine derivatives of CA-4 and evaluated these derivatives for their scavenging properties towards radicals such as DPPH, NO, SOR and H_2_O_2_. All of the synthetized compounds revealed good to perfect efficacy against DPPH, NO and SOR radicals, while they showed good to moderate hydrogen peroxide radical scavenging activity (between 36.55 and 47.17%) [29].

### 3.2. Anti-Inflammatory Activity

Inflammation and cancer are closely related, and many anti-cancer drugs are known to also be usable to treat inflammation. It has been demonstrated by several studies that cancer cells express cytokines and chemokines and that their respective receptors have an important effect on angiogenesis, cell migration and metastasis [30]. The fact that combretastatins cause mobilization in the form of endothelial cells, impaired intercellular adhesion and increased permeability of the vascular wall can change the course of inflammatory processes [31].

In a study, the effects of CA-4 and new, *cis*-restricted analog CA-432 on the NF-κB signal pathway in T cells were investigated. Western blotting and NF-κB reporter gene activity were used for assessment. CA-4 and CA-432 both inhibited TNF-a-stimulated NF-κB inducing through IκBα disruption and reduction of p65 nuclear translocation and inhibition of NF-κB reporter gene activity [32].

**Figure 5:**
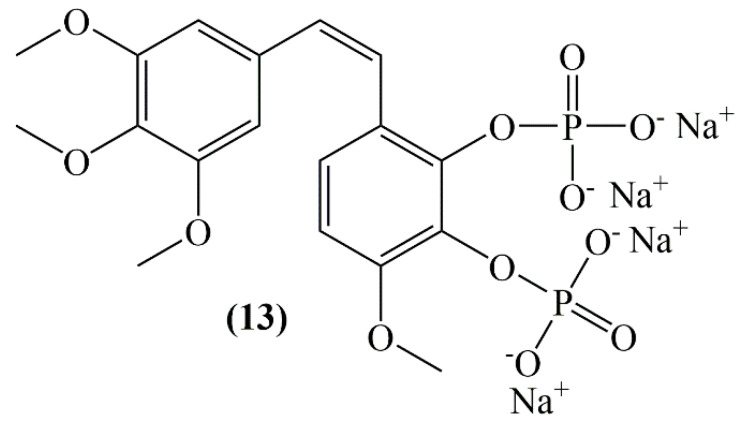
Structure of combretastatin A-1 phosphate (**13**).

The effects of combretastatin A-1 phosphate (CA-1P) (**13**, Figure 5) towards tumor-associated macrophages were investigated in hepatocellular carcinoma in mice. As a result of western blot assays of Raw 264.7 cells, combretastatin A-1 phosphate down-regulated the proteins related to the pathway of p-AKT, Mcl-1 and Wnt/β-catenin, comprising GSK-3βSer9 and β-catenin. With this study, CA-1P has been successfully proved to have the power to affect both cancer cells and microenvironment [33].

**Figure 6:**
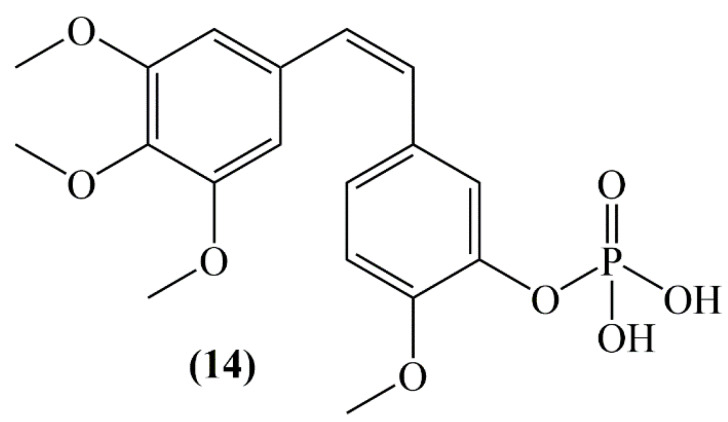
Structure of combretastatin A-4 phosphate (14)**.**

In a study of an ulcerative colitis mouse model, the effects of combretastatin-A4 phosphate (CA-4P) (**14**, Figure 6) were evaluated in inflammation induced by dextran sulfate sodium (DSS). According to the results of the study, CA-4P (11 mg/kg/d, i.p.) significantly reduced body weight, colorectal length and increased disease activity index in DSS-induced mice. mRNA expression of TNF-α, IL-6 and IL-1β in colon tissue was found higher in DSS + CA-4P group than DSS-stimulated ulcerative colitis model group [34].

A study examining the mechanisms underlying the effects of 4,5-diarylimidazole brimamin which is water-soluble and a derivative of the vascular-disrupting agent CA-4, on endothelial and carcinoma cells has been conducted. In BxPC-3 pancreatic cancer cell and triple negative breast cancer cell (MDA-MB-231), it was concluded that brimamin activated apoptosis at nanomolar concentrations by inhibiting NF-κB stimulation [35].

Davydova et al. evaluated the effect of the combretastatin analogs they synthesized in histamine and concanavalin A-induced inflammation mice models. In the histamine inflammation model, piperidine and morpholine fragments did not exhibit anti-inflammatory activity, pyrrolidine, piperidine and morpholine residues containing β-aminovinylketones reduced the inflammation by an average of 1.5-fold, compared to control group. In the inflammatory model induced by concanavalin A, compounds carrying piperidine and morpholine fragments, increased inflammation relative to the control. Other combretastatin derivatives also had no significant effect on concanavalin A-induced edema [31].

### 3.3. Antimicrobial and Leishmanicidal Activities

In the light of ethno-botanical information it is known that *Combretum* species are traditionally used in the treatment of diseases such as syphilis, conjunctivitis, diarrhea, toothache, peptic ulcer and dysentery [36]. In line with this information, the antibacterial activities of many *Combretum* species were evaluated [37], while the antibacterial activities of combretastatins or their synthesized derivatives were examined in a limited number of studies.

Eloff et al. reported that combretastatin B5, isolated from *C. woodii* leaf extract, revealed strong bactericidal effects against *Staphylococcus aureus,* with a MIC value of 16 mg/mL and a weak effect towards *Pseudomonas aeruginosa* and *Enterococcus faecalis* with MIC values of 125 mg/mL [37].

Gonzales et al. evaluated the antiviral and antifungal activities of combretastatin synthesized from gallic acid and three combretastatin-derived analogs. Yeast *Candida parapsilosis*, *C. krusei, C. tropicalis, C. albicans,* and the fungi *Aspergillus fumigatus, A. flavus, A. terreus, Fusarium oxysporum* and the dermatophytes *Trichophyton rubrum, T. mentagrophytes* were utilized in the antifungal activity screens. The combretastatin-based derivative **11** and CA-4 demonstrated moderate antifungal activity towards the dermatophytes *T. rubrum* and *T. mentagrophytes*. The MIC values of CA-4 against *T. rubrum* and *T. mentagrophytes* are found to be 19.8 ± 6.5, 15.8 ± 6.5 μg/mL, respectively. No activity of the compounds against *C. parapsilopsis, C. krusei, C. tropicalis, C. albicans, A. flavus* and *A. oxysporum* was observed at doses of less than 100.0 μg/mL. CA-4 and hybrid **9** revealed activities against HHV-1, and HHV-2. The antiviral activities of CA-4 against HHV-1, and HHV-2 are 50 and 25 μg/mL, respectively [38].

**Figure 7:**
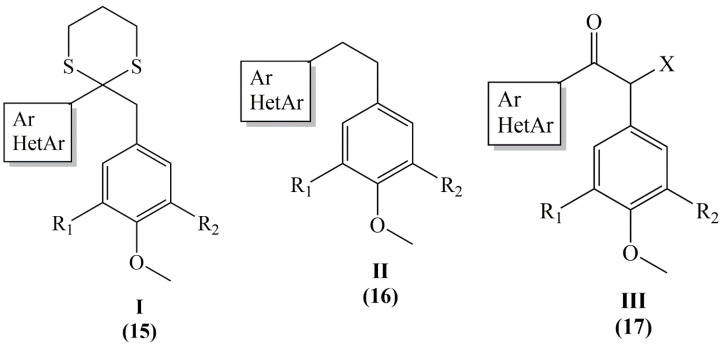

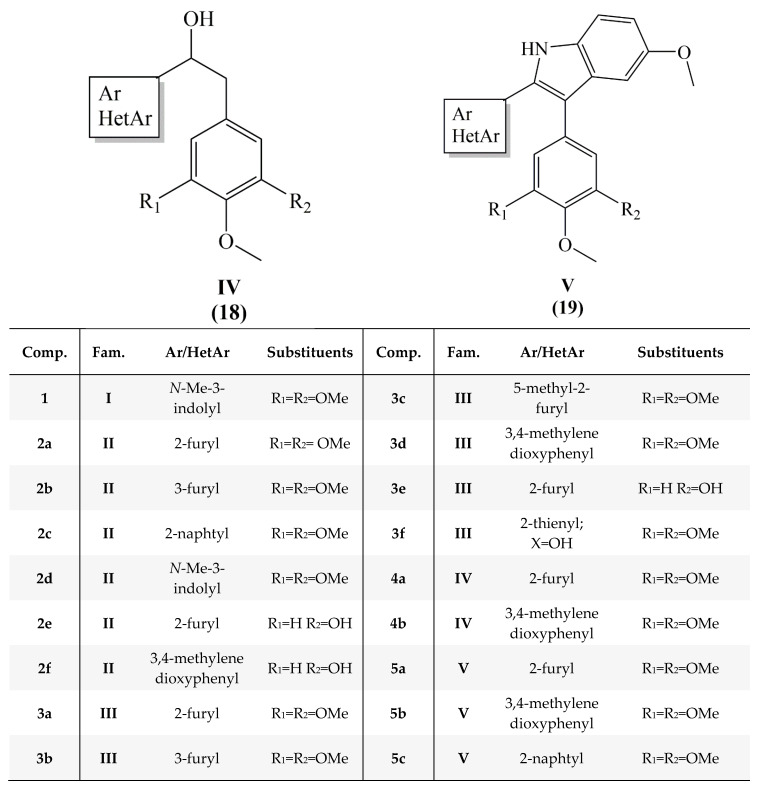
Structure of dithianes (Series I), ethane (series II), ethanones (series III), ethanol (series IV) and indole (series V) derivatives of combretastatin.

Combretastatins and heterocombretastatins have been tested in vitro on parasitic cultures of Leishmania species. Dithianes (series I), ethane (series II), ethanones (series III), ethanol (series IV) and indole (series V) derivatives named as 1 (**15**), 2a–f (**16**), 3a–f (**17**), 4a–b (**18**) and 5a–c (**19**) (Figure 7). When 2a, 2d, 3a, 3e, 3d and 5a were incubated for 48 h with L. amazonensis, L brasiliensis, L. donovani, lysis at 10 µg/mL reached 90% of the total promastigotes, while at 50 µg/mL total parasite lysis was observed. Compounds containing a 3,4,5-trimethoxyphenyl group was found to be better than compounds containing a 3-hydroxy-4-methoxyphenyl group between the compounds **2a**–**2e** and **3a**–**3e**, similarly the 2-furyl group results better than the 2-naphthyl group (**2a/2c**) and close to the 3,4-methylenedioxyphenyl group (**2e/2f**). 2-furyl, 3,4-methylenedioxyphenyl and 2-naphthyl residues containing compound **5a** was found to have most effective leishmanicidal activity [39].

### 3.4. Vascular Effects

The indirect death of tumor cells by targeting tumor vasculature paves the route for cancer treatments. In this approach the formation of new blood vessels (angiogenesis) is blocked or the existing vasculature is targeted [40]. The tumor vasculature targeting effects of combretastatins and their synthetic analogs have been demonstrated by numerous studies in various animal models.

In one study, the prodrug CA-4 has been shown to cause vascular closure at low doses. Short-term drug administration showed long time antiproliferative/cytotoxic effects towards proliferating endothelial cells. Vascular closure in human breast cancer models subsequent to drug application continued with a 93% reduction in functional vascular volume, 6 h after drug administration and with the corresponding histology coherent with hemorrhagic necrosis for the next 12 h [41].

Chaplin et al. showed in their study that combretastatin stopped blood flow more commonly in tumors than healthy tissues. According to histological evaluations, more than 90% of the vessels were reported to be dysfunctional at a combretastatin dose of 100 mg/kg (i.p.) after 6 h [42].

The combination of CA-4P and inhibitor of nitric oxide synthase (*N*-ω-nitro-l-arginine methyl ester) strengthened the influence on tumor tissue. The results of the study showed that nitric oxide preserve normal tissues against the harmful vascular influence of CA-4P. A rise in arterial blood pressure and a very high decline in tumor blood flow were observed 1 and 6 h later CA-4P application at 100 mg/kg (ip) concentration. Promising results for utilization of CA-4P as a tumor vascular targeting agent were elucidated by this study [43].

In a different study, a murine endometriotic lesion model induced by syngenic transplantation into the dorsal skin fold chambers of the endometrium, mice were administered ip 80 mg/kg CA-4P 6 days later. CA-4P injection caused selective vascular damage without disrupting the microvascular system around the endometriotic lesion. When evaluated together with the control groups, it has been observed that CA-4P applied lesions decreased the functional capillary density and blood perfusion after 2 h [44].

Mice bearing MHEC5-T hemangioendothelioma were administered only one concentration of OXI4503 (CA-1P) at 100 mg/kg and the tumor cell killing mechanism, vascular functional and morphological differences were examined. The blood flow in the tumor was measured with microsphere fluorescence and a 50% reduction was observed in 1 hour and achieved a maximum of 6-24 h after drug application [45].

By displacing the ethylene bridge with a 1,4-diaryl-2-azetidinone (β-lactam) ring in the combretastatin structure, *cis-trans* isomerization was prevented and a group of *cis* restricted CA-4 derivatives was synthesized by Nathwani et al. The effect of synthesized β-lactam compounds on tumor vascularization and its effect on tumor cell migration were investigated. It has been determined that CA-104 and CA-432 β-lactam compounds exhibited their anti-angiogenic effects in MDA-M′-231 breast adenocarcinoma cells by decreasing the release of vascular endothelial growth factor [46].

**Figure 8:**
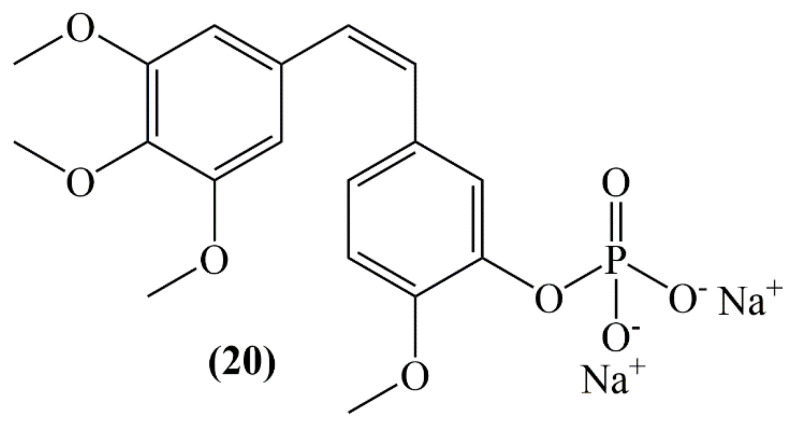
Structure of combretastatin A-4 disodium phosphate (**20**).

Study investigating the competency of combretastatin A-4 disodium phosphate (CA-4-DP) (**20**) (Figure 8) to stimulate vascular effects in mouse breast carcinoma, the anti-tumor response was investigated by combining CA-4-DP with the anticancer drug cisplatin. CA-4-DP (250 mg/kg) was found to markedly reduce tumor perfusion 30 min after injection and this effect lasted for a few hours, and after 24 h a return to normal was observed. Administration of the drug 1 h after cisplatin injection resulted in a bigger tumor response than for the additive only [47].

The important clinical study results obtained with CA-4P have elucidated the anti-tumor and anti-vascular effects of its analogue combretastatin A1 phosphate. This compound like CA-4P leads to an in vitro degradation of the tubulin cytoskeleton in human umbilical vein endothelial cells at the same doses and after similar exposure time. It was reported that administration of combretastatin A1 phosphate at 150 mg/kg dose to vascularized murine colon adenocarcinoma (MAC 29) caused a strong decrease in vascular volume after 2 h [48].

CA-4P has been indicated to selectively target endothelial cells and induce remission of vascularization of unstable new tumor formations. The tumor vascular process of action has been described as an endothelial cell-specific junction molecule that rapidly prevents the molecular involvement of the vascular endothelium cadherin in vitro and in vivo. In addition, CA-4P has been reported to intensely inhibit endothelial cell migration and capillary tube formation by inhibiting the VE-cadherin/β-catenin/Akt signaling pathway, thereby causing vascular collapse and tumor necrosis [49].

Fruytier et al. investigated the effect of discontinuation of blood flow caused by CA-4 on the intake of gemcitabine in mouse hepatocarcinoma. A markedly reduction in perfusion-related parameters (K trans and Vp) in the treatment group was observed 2 h after CA-4 application compared to control group. The cessation of blood flow has been proven by a histological study. As tested in a different assay with ex vivo fluorine nuclear magnetic resonance spectroscopy, total gemcitabine intake has been reported to be markedly in minimum amounts in treated tumors. The important point of the study is that blood flow shutdown stimulated by VDAs can negatively affect the dispensation of small molecular structure cytotoxic drugs in tumors [50].

The effects of CA-4, which inhibits retinal neovascularization in the murine model of ischemia-induced proliferative retinopathy, were evaluated by Griggs et al. CA-4 led to a concentration-dependent neovascularization inhibition without significant adverse effects, and the lack of vascular abnormalities or impaired retina neovessels of mice treated with CA-4 proved to be influenced by the anti-angiogenic mechanism [51].

In order to evaluate the activity of CA-4P in ocular neovascularization, models were set as mice with overexpression of vascular endothelial growth factor in the retina and mice with choroidal neovascularization due to laser-stimulated rupture of the Bruch membrane. In the first experimental model, the total area of neovascularization per retina was 0.01415 ± 0.00432 mm^2^ in mice treated with 4 mg/kg CA-4P and in the vehicle-treated group, this area was found to be 0.06112 ± 0.03436 mm^2^. In the second experimental model, the group that received CA-4P at a dose of 75 or 100 mg/kg by daily intraperitoneal injection showed a marked reduction in choroidal neovascularization in the rupture sites compared to the group with vehicle injected mice. According to the results of the study, CA-4P has been reported to be an effective agent in both prevention and treatment of ocular neovascularization [52].

In the hyperplastic thyroid model, goiter was induced in mice and the effects of CA-4 as a tumor-specific anti-vascular agent were evaluated. While *cis*-CA-4 inhibited primary tumor growth and metastatic development, *trans*-CA-4 did not affect primary and secondary tumor growth. *cis*-CA-4 has been shown to induce widespread microthrombus formation in rapidly growing vasculature [53].

Tumor vascular effects of CA-4P at doses of 30 and 100 mg/kg were monitored online by intravital microscopy before and after treatment of rats. The combination of CA-4P and N-(ω)- nitro-L-arginine systemic nitric oxide synthase (NOS) inhibitor has also been evaluated for its effects. A rapid decrease in tumor blood flow was found after CA-4P treatment and it was reported that the red cell velocity decreased to < 5% of the initial value after 1 h. Although administration of the NOS inhibitor alone caused a 50% reduction in red cell speed, the effect of the combination with CA-4P on mean arterial blood pressure was not significantly different from *N*-(ω)-nitro-L-arginine alone [54].

Some triazole (compound **1**), tetrazole (compound **2**) combretastatin analogs and compound **3**, where CA-4’s ring 3′-hydroxy, 4′-methoxyphenyls was replaced with a thiazolylphenylamine moiety, were tested for their anti-angiogenic effects in HUVEC and in vivo in chick chorioallantoic membrane assay (CAM), and in a syngeneic tumor mouse model. All compounds revealed moderate activity on the growth of HUVEC cells at concentrations below 200 nM and increased permeability of HUVEC cells. In CAM analysis, compounds **2** and **3** effectively responded to the strong angiogenic response caused by bFGF, even at the lowest concentration used (1 pmol/egg). In mouse model, after the intraperitoneal injection of compounds **1**−**3** (30 mg/kg), microvascular density was strongly reduced [55].

Although the poor water solubility of combretastatin A-4 is a problem in drug development, a water-soluble analog combretastatin A-4 phosphate (Zybrestat) has received orphan drug status from the U.S. Food and Drug Administration (FDA) and the European Medicines Agency (EMA) for use in the treatment of thyroid cancers. The vascular disrupting agent, Zybrestat prodrug, is indicated for the treatment of anaplastic thyroid cancer and, potentially other solid tumors. The data obtained from a phase II study examined the clinical effects of Zybrestat, paclitaxel and carboplatin combination, which have been tried in patients with advanced malignancies including anaplastic thyroid cancer. Compared to treatment with paclitaxel and carboplatin alone, Zybrestat has been shown to possess strong anti-tumor activity. CA4P, which eliminates large areas of central weak vascularized parts of solid tumors known to be resistant to many classical anticancer treatments, has the potential to complement existing treatments [56]. Both FDA and EU also granted orphan drug designation for OXi4503 in addition to Fast Track status for the treatment of acute myeloid leukemia. The *cis*-olefin structure of combretastatin causes in vivo stability problems, and many analogs synthesized in order to overcome this problem are also under trial in clinical studies [57].

### 3.5. Tubulin Binding Activity

Cancer is one of the largest causes of disease-related deaths worldwide. It is defined as the irregular differentiation, proliferation and accumulation of cells in an organism. In medicine, it is called a malignant neoplasm that seriously threatens humans’ life. There are actually three main methods of treating cancers, including anticancer drug treatment, surgery, and radiation therapy. Treatment with anticancer drugs can be focused on malignant tumors which occur at different stages in different parts of a body. Anticancer drugs are classified into three groups according to the kinetics of cell proliferation. Drugs in the first group interfere with nucleic acid biosynthesis and transcription of DNA and affect the structure and function of DNA. The second group of drugs inhibit the activity of protein while the third group affect the endocrine system of the body. The second group of anticancer drugs is mostly common clinically used drugs because of their good drug efficacy and low toxicity. The crucial target for this group of drugs is tubulin. These drugs can bind to tubulin and interfere with the microtubule dynamics of tubulin [58].

Microtubules, which are composed of α/β tubulin dimers, form the microtubule filament system of eukaryotic cells. These α/β heterodimers are the main building material for the microtubular cytoskeleton. A microtubule is a tube made from parallel linear polymers (protofilaments) where the heterodimers are mounted in a polar pattern, head to tail. These protofilaments constitute the cylindrical and helical microtubule wall [59,60]. Microtubules have various important functions in eukaryotic cells such as cell division (the formation of the mitotic spindle), cell motility and migration, intracellular transport, cell signaling and maintaining cell polarity [58,59,61].

**Figure 9:**
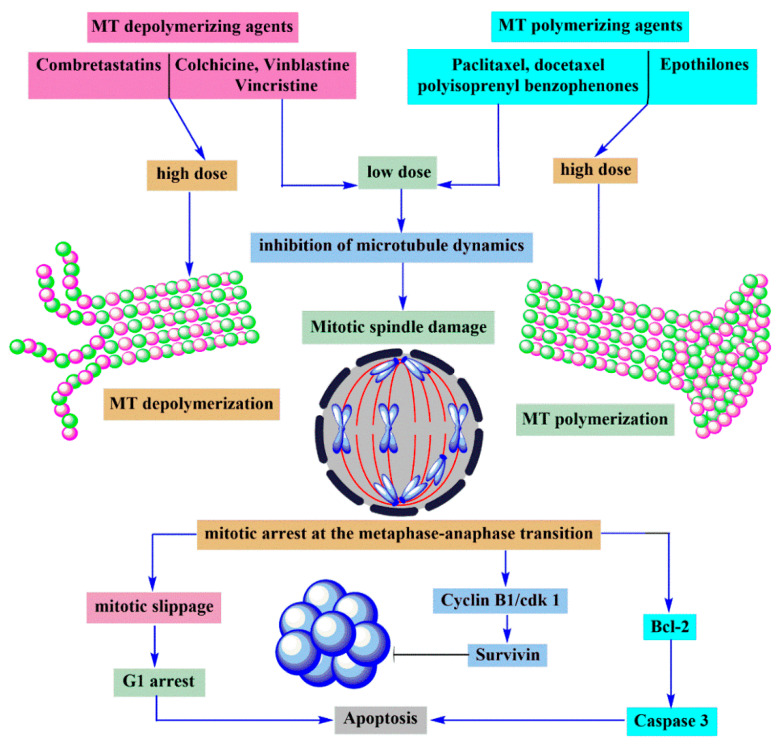
Schematic describing proposed mechanism of action of the different periods of microtubule targeting agents. At high dose combretastatins, colchicine, vincristine and vinblastine induce microtubule depolymerization, while paclitaxel, docetaxel, polyisoprenyl benzophenones and epothilones promote microtubule polymerization; at low dose, all microtubule-binding agents inhibit microtubule dynamics, prevent the proper alignment of chromosomes at the metaphase plate and segregation of chromosomes in anaphase, leading to mitotic arrest and apoptosis.

Microtubules are thought to be available targets for cancer chemotherapy. The microtubule targeting agents (MTAs) bind to the complex three-dimensional surface of the tubulin polymer and exhibit antiproliferative activity. MTAs exert their effects as microtubule stabilizers or polymerizing agents [59,61,62]. Paclitaxel, docetaxel, vinblastine, vincristine, vinorelbine, and vindesine are highly effective anticancer agents acting as microtubule-targeting [62]. Paclitaxel, docetaxel and the polyisoprenyl benzophenones effect as polymerizing agents while colchicine, vinblastine, and vincristine as depolymerizing agents [59]. In addition to these natural products, several natural products such as combretastatins, epothilones, dolastatins, and 2-methoxyestradiol are in clinical trials for cancer chemotherapy [62] (Figure 9).

Microtubule targeting agents (MTAs) induce the apoptosis of cancer cells. Generally, most of MTAs arrest the cell cycle at the G2/M stage. Li et al. suggested that hyperphosphorylation of Bcl-2 and/or Bcl-xL by CDC2 or JNK, phosphorylation of BAD on serine 128 by CDK1, and Bim activation by JNK could be the mediators linking MTA-induced mitotic arrest and apoptosis [59].

Combretastatins bind at the colchicine binding site on the β-tubulin subunit of tubulin and cause depolymerization of tubulin, which is known to be separate from the vinca binding site [18,59,62]. Recent studies on some types of MTAs revealed that colchicine site binding drugs such as the combretastatins can inhibit the formation of new blood vessels in cancer tissue selectively as vascular disrupting agents (VDAs) [61,63]. Among the combretastatins, CA-4 is the most active compound, with strong antiproliferative and antiangiogenic activities. Phase II clinical trials for the treatment of advanced anaplastic thyroid cancer, pathologic myopia and polypoidal choroidal vasculopathy have been completed for CA-4P, a prodrug of CA-4. Moreover, CA-4P is in continuing phase III clinical trials for anaplastic thyroid cancer with carboplatin/paclitaxel combination [62].

CA-4 has a potent cytotoxic effect against many human cancer cell lines including multidrug resistance (MDR) cancer cells in vitro while not in vivo due to its low solubility and the instability of its *cis* configuration. Therefore, phosphate prodrugs of combretastatins has been designed to circumvent low water solubility [59].

In solid tumors, CA-4P exhibited antiproliferative effect on the inner part of the tumor tissue resulting to leave some of viable tumors at the periphery. Combination of CA-4P with other therapies is preferred to prevent this condition, thus more effective therapy is provided [59].

Although CA-4 has antiproliferative, antiangiogenic and antivascular activities and is a promising clinical candidate, it has a poor bioavailability due to its low water solubility, short biological half-life and *cis-trans* isomerization [62]. Therefore, many CA-4 modifications have been made in order to design new and more potent compounds with improved in vivo therapeutic profiles. Previous studies on structure-activity relationship of CA-4 showed that the presence of cis-configuration double bond in the C-2 bridge is necessary for high cytotoxic and antitubulin activity, efficient binding to tubulin is possible with trimethoxybenzene moiety and 3,4,5-trimethoxysubstituted A ring and the 4-methoxy-substituted B ring are also important for cytotoxicity and binding at the colchicine site on the β-tubulin [60,64].

**Figure 10:**
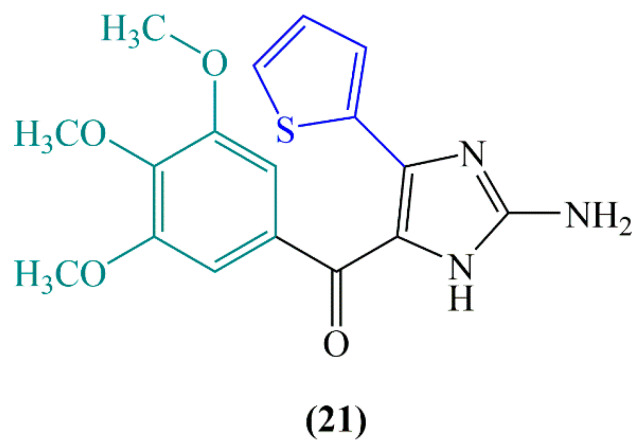
Structure of combretastatin inspired 2-aminoimidazole (**21**).

Replacing the olefinic bridge with heterocyclic or carbocyclic rings and bridging functional groups has been a beneficial approach for inhibition of metabolic degradation and maintenance of the cis conformation of the structure that substitution of olefinic bond of CA-4 (**1e**) with a 2-aminoimidazole enhances tubulin binding activity (**21**, Figure 10).

**Figure 11:**
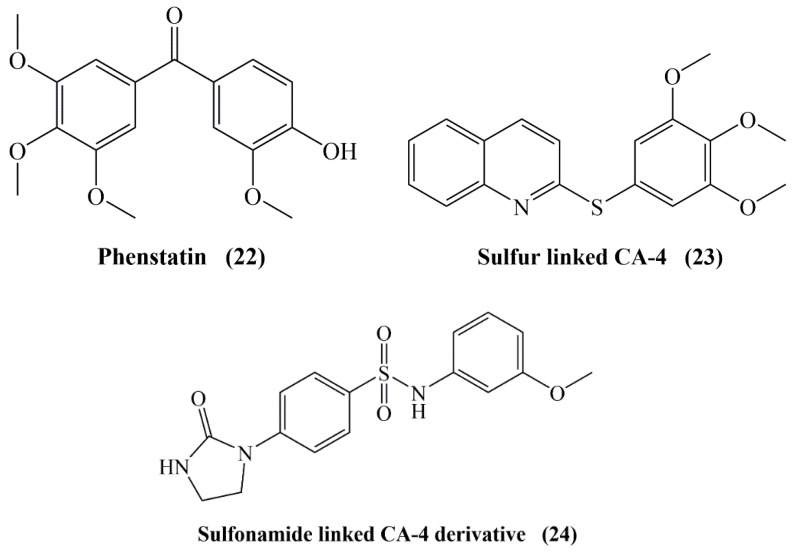
Structure of combretastatin by replacement of the ethenyl bridge of the stilbene moiety with phenstatin, sulfide and sulfonamide.

Moreover, it can increase water solubility and induce drug like properties [62]. However, replacement of the ethenyl bridge of the stilbene moiety with a stable keto group (phenstatin, **22**), sulfide (**23**), ether, and sulfonamide (**24**) (Figure 11) provides improved physical properties. Accordingly, various derivatives such as CA-4P, CA-4NH_2_HCl (hydrochloride salt form of CA-4) and CA-4NH-Ser (serine prodrug form of CA-4) developed by the replacement of substituents at the *meta* position of the B ring of CA-4 have showed significant antimitotic activity with improved bioavailability [60].

The carbonyl oxygen in new agents such as aroylindole and diarylketone chalcone was found to increase the antiproliferative ability. The 2-aminoimidazole-carbonyl derivative of CA-4 that possesses a thiophene as ring B was determined to be significantly more effective than CA-4 against lung carcinoma cells. Furthermore, this derivative of CA-4 exerts more selective cytotoxicity against breast cancer cells than its parent compound CA-4 [62]. However, studies on exploring different alternatives of 3-hydroxy-4-methoxyphenyl ring (B-ring) of CA-4 (**1d**) have revealed indole or naphthalene analogues with highly potent activities but with reduced water solubilities [61].

The addition of polar groups and masking them as intramolecular hydrogen bonds is a popular technique applied to improve the solubility of drugs [61]. However, if the polar groups are positioned in the target’s hydrophobic regions, this strategy may be inconvenient as it is the case with the colchicine site. Therefore, a novel alternative strategy called masked polar group incorporation (MPGI) have been performed in the study of Gonzalez et al. in order to mask polar groups from the outside leading to binding at low polar regions of the target while increasing the intrinsic aqueous solubility. In this method, polar groups are introduced with vicinal bulky substituents. For this reason, nitrogen atoms are placed on phenyl rings acting as the masking groups to form pyridines within the context of dimethylaminocombretastatin and isocombretastatin analogues. The new compounds synthesized showed improved water solubility and highly significant antiproliferative activity against several cancer cell lines [61].

### 3.6. Antiproliferative Activity

**Figure 12:**
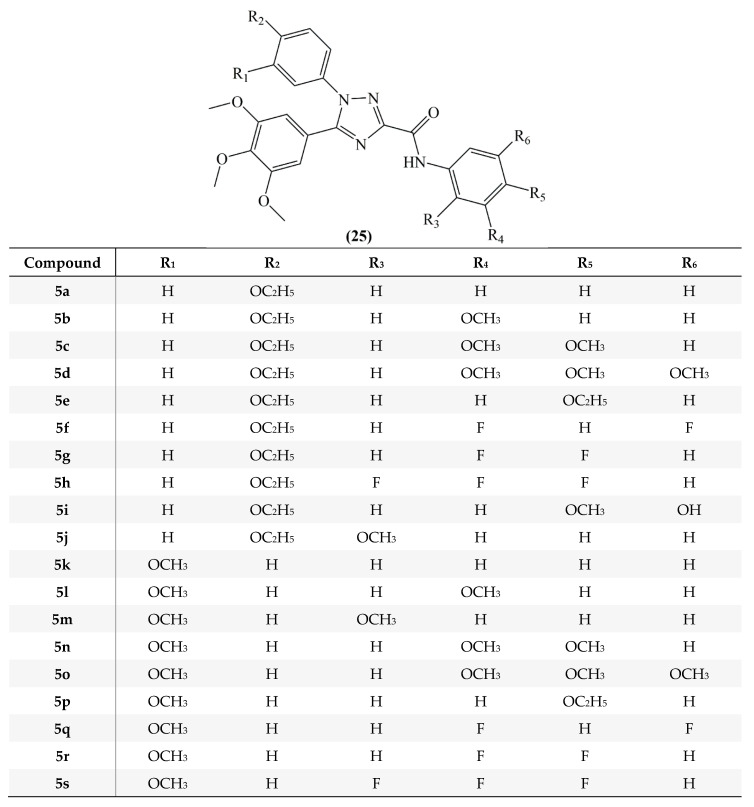
Structure of *N*-acylhydrazone analogs designed as comretastatin A4 derivatives (compounds **5a**–**5s**).

The selective cytotoxic effects of *N*-acylhydrazone analogs designed as combretastatin A4 derivatives and their antiproliferative effects against human lymphocyte cells were evaluated. Antiproliferative effects of compounds **5a**–**r** (**25**, Figure 12) were evaluated against HL-60 (human leukemia), SF-295 (human glioblastoma), MDA-MB435 (melanoma), PC3M (prostate cancer), OVCAR-8 (ovaries adenocarcinoma), NCI-H258M (pulmonary bronchio-alveolar carcinoma), HCT-8 (adenocarcinoma) and human lymphocytes. Except compounds with bulkier groups attached to the imine named as **5i**, **5j**, **5k** and **5n** analogs, the remaining compounds containing hydrophobic substituents on ring B, **5l**, **5m**, **5o**, **5p**, **5q** and **5r**, showed moderate to strong antiproliferative activity and the IC50 values were found between ≤ 18 µM and ≥ 4 Nm. Compounds with oxygenated substituents at the ring B **5a**, **5c**, **5d**, **5e**, **5f**, **5g** and **5h** were found to have less cytotoxic activity than CA-4. Analogs **5b**, **5l**, **5m**, **5o**, **5p**, **5q**, **5r** showed cytotoxic effects against human lymphocytes, similar to the precursor compound CA-4. Also CA-4 has been proven to be a non-selective cytotoxic agent in this study [65].

**Figure 13:**
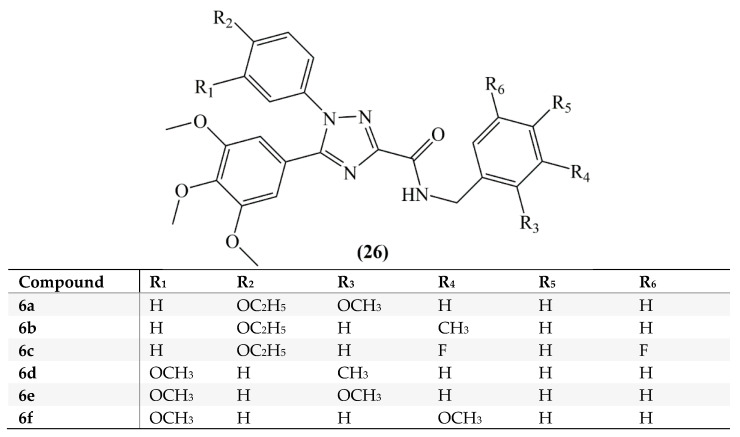
Structures of compounds **6a**–**6f**.

Mustafa et al, synthesized *cis*-restricted analogs of CA-4 containing 1,2,4-triazoles. Compounds were divided into two series. In the first series, for analogs **5a**–**5j** the A ring used was a 3,4,5-trimethoxyphenyl moiety, the B ring a 4-ethoxyphenyl moiety and for analogs **5k**–**5s** a 3-methoxyphenyl moiety was used. In addition, in order to improve binding with the colchicine binding region of the tubulin, an extension was provided to the structure by adding the carboxamide group attached C ring. In the second series for analogs **6a**–**6f** (**26**) a benzyl group was added to the structure (Figure 13). Hepatocellular carcinoma (HepG2) cells and leukemia (HL-60) cells were used in the antiproliferative activity tests. According to the results for HepG2 cells, analogs **5q**, **6a**, **6b**, **6c**, **6e**, and **6f** revealed cytotoxic effect better than CA-4 and the IC_50_ values were found between 0.62 and 1.90 Mm. Compounds of series 2 revealed strong antiproliferative activity in HepG2 cells. Among the second series of compounds analog **6a** exhibited an antiproliferative activity near that of CA-4 against HL-60 cells [66].

**Figure 14:**
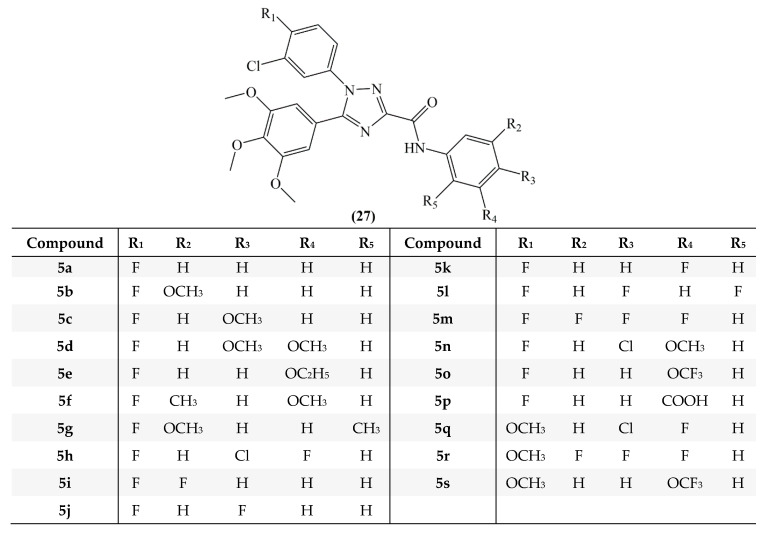
Structure of 1,2,4-triazole-3-carboxamide analogs of combretastatin.

In a different study of Mustafa et al, 1,2,4-triazole-3-carboxamide analogs of combretastatin were synthesized (**27**, Figure 14) and studied for their antiproliferative activity towards HepG2, HL-60, and MCF-7 (breast cancer) cell lines. On HepG2 cells compounds **5b, 5o**, and **5r**, on HL-60 cells compounds **5b, 5f, 5i, 5k, 5o**, and **5q** and on MCF-7 cells compounds **5b, 5q**, and **5s** revealed strong inhibitory activity. In the study, it was reported that replacing the hydroxyl group with chlorine in the meta position of the B ring preserves the activity of the analogs and using fluorine instead of the methoxy group strengthens the effectiveness [67].

Mainly piperazine-derived compounds were obtained by making modifications to the stilbene linking group of a new series of compounds with a CA-4 core structure. All synthesized analogs were tested against MCF-7 cells. Two amino-containing derivatives **4q**, **4x** and hydroxyl derivative **4m** exhibited strong activity with 130 nM, 83 nM and 190 nM IC_50_ values, respectively [68].

Carr et al. synthesized β-lactam derivatives of combretastatin A and tested their antiproliferative activity using two breast cancer cell lines, the ER dependent MCF-7 human breast cancer and the ER independent MDA-MB-231 human breast cancer cell lines, using the MTT (tetrazolium)-based viability assay. The 3,4,5-trimethoxyphenyl ring located at the N-1 position was found to be much more potent in both cell lines (with IC_50_ values of 0.010 and 0.017 uM on MCF-7 and 0.047–0.054 uM on MDA-MB-231) compared with the compounds with a 3,4,5-trimethoxyphenyl ring is located at the C-4 position (2.96–4.04 uM on MCF-7 and 13.03–3.19 on MDA-MB-231). Replacement the β-lactam group with the thione resulted a reduction in the antiproliferative activity and it is estimated that this could be due to the difference in lipophilicity when compared with the containing ones [24].

Acylhydrazone, chalcone and amine-bridged derivatives of CA-4 were synthesized for comparison of their activity towards tubulin. Amongst the metabolites with acylhydrazone bridge with a benzyl at the indole-N position was determined as the most potent antiproliferative agent against several cancer cell lines (IC_50_ values between 0.08 and 35.6 μM) with lower cytotoxicity activity on normal human cells as well [69].

In order to inhibit *cis* to *trans* isomerization of CA-4 analogues, the *cis* double bond was replaced with a pyridazine ring and novel 5,6-disubstituted pyridazin-3(2*H*)-one derivatives were synthesized. These derivatives were tested on four different human cancer cell lines (HL-60, MDAMB-435, SF-295 and HCT-8), however, no remarkable activity was seen on any of these cell lines or tubulin itself [70].

*Cis/trans* isomerization of CA-4 is responsible for this vascular targeting agents’ inactivation. For the prevention of this inactivation and to improve the antiproliferative activity several azetidin-2-ones substituted at positions 2, 3 and 4 of the azetidinone ring scaffold were generated by using Staudinger and Reformatsky reactions. The 3-(2-thienyl) analogue **28** and the 3-(3-thienyl) analogue exhibited the highest activity in MDA-MB-231 breast cancer and NCI-60 cell lines. Moreover, no significant toxicity was seen in normal murine breast epithelial cells. Larger group substitutions at position 3 such as 3-naphthyl derivatives displayed lower antiproliferative activity [71].

There are many studies related to the *cis/trans* isomerization of CA-4. Primarily, instead of the double bond between the A and B rings, various heterocyclic rings were added to the structure to inhibit this isomerization [72,73]. A series of derivatives starting from AC-7739 and AC-7700, were developed. Especially, pyrazole and tetrazole rings instead of the double bond in the linker were synthesized. Compounds with a pyrazole ring displayed antiproliferative activity with an IC_50_ value of 8.4 nM against colon-26 adenocarcinoma cell line and inhibited tubulin polymerization with an IC_50_ value of 3 μM. Similarly, compound **2** with a tetrazole ring showed the cytotoxicity with 7.2 nM and inhibited tubulin polymerization with an IC_50_ of 2 μM [72,73].

Accordingly, Nakamura and colleagues synthesized new series of CA-4 analogues by placing double bonds with a silicon atom mimicking the same distance between two phenyl rings [74]. The average length of a C-C bond was measured as 1.53 Å, while the new compounds length of a Si-C bond was measured as 1.89 Å [75]. The length among two phenyl rings in the original compound CA-4 was 3.0 Å, and the distance between two phenyls in new synthesized compound with silicon is calculated as 3.3 Å. This compound inhibited the tubulin polymerization by 51% at a concentration of 30 μM and showed cytotoxicity with an IC_50_ value of 7 nM in the human breast cancer (MCF7) cell line. The critical part is this new compound is found to be more stable than CA-4 [74].

Along with the instability of CA-4 due to the *cis/trans* isomerization, poor solubility is also an important defect of this compound. Synthetic derivatives were produced in order to improve stability, solubility and therapeutic activity. Molecules with 3,4,5-trimethoxy aromatic rings at position 1 and a variety of substitution patterns at positions 3 and 4 of the β-lactam ring were tested against three adenocarcinoma-derived colon cancer cell lines (CT-26, Caco-2 and the CA-4 resistant cell line, HT-29). All metabolites showed improved activity against CT-26 and Caco-2 cells even at the nanomolar range. Substitution of the bridge between the rings of CA-4 with a β-lactam ring together with the aforementioned aryl substitutions improved the therapeutic efficacy of CA-4 up to 300-fold in combretastatin refractory HT-29 cells. combretazet-3 (CAZ-3, 4-(3-hydroxy-4-methoxy- phenyl)-3-(4-hydroxyphenyl)-1-(3,4,5-trimethoxyphenyl)azetidin-2-one) displayed chemical stability along with the higher bioactivity compared with CA-4. Moreover, this compound also demonstrated significant tumor inhibition in a murine model of colon cancer [76].

Combretastatin A-4 prodrug has been known as a antivascular agent in various rodent tumor models. Boehle and colleagues, investigated the activity of this agent on human non–small cell lung cancer (NSCLC). In vitro studies revealed the time and dose dependent antiproliferative activity, probably by disruption of microtubule assembly. Furthermore, CA-4PD remarkably inhibited the growth of subcutaneously induced lung cancer by antivascular effect [77].

In another study, CA-4 was integrated with sulfonyl piperazine scaffolds as a one molecule platform and tested for antiproliferative activity against a panel of human cancer lines cell lines, namely lung (A549), mouse melanoma (B16F10), breast (MDA MB-231and MCF-7) and colon (HCT-15) by MTT assay. Amongst the synthesized derivatives, the compound (*E*)-3-(4-chlorophenyl)-1-(4-((4-chlorophenyl)sulfonyl)piperazin-1-yl)-2-(3,4,5-trimethoxyphenyl)prop-2-en-1-one (**5a,b**) showed potent antiproliferative activity on selected cell lines with IC_50_ values between 0.36 and 7.08 μm [78].

Greene et al. synthesized series of CA-4 metabolites incorporating a 3,4-diaryl-2-azetidinone (β-lactam) ring to improve stability and activity. The synthesized β-lactam CA-4 analogs demonstrated significant antitubulin, antiproliferative, and antimitotic effects in human leukemia cells [79].

The antitumor activity of new synthesized 2-amino and 2′-aminocombretastatin derivatives were evaluated. Amongst, several of them showed very potent antiproliferative activity as inhibitors of tubulin polymerization with IC_50_ values of 1.6, 1.7, and 1.8 μM even higher than colchicine and comparable to CA-4. They exhibited antiproliferative activity with IC_50_ values of 11 to 44 nM in several human cell lines from different organs. SAR studies revealed that a NH_2_ substituent at the 2-position of either ring A or ring B probably plays an important role in the bioactivity of this series of compounds [80].

By oxidation of diarylalkynes promoted by PdI_2_ in DMSO, 14 novel benzyl derivatives related to CA-4 has been synthesized. Several benzyls showed excellent antiproliferative activity at the nanomolar level (20–50 nM) on four human tumor cell lines. Several of them displayed the better cytotoxicity against HCT116 were next evaluated against three different cancer cell lines. All metabolites exhibited significant cytotoxic activity (about 30 nM) against the human colon carcinoma (HCT116), chronic myelogenous leukemia (K562), non-small lung human carcinoma (H1299), human breast cancer (MDA-MB231) cell lines [81].

New CA-4 derivatives with a 3’-*O*-substituted carbonic ether moiety synthesized. These derivatives showed potent antitumor activity against MDA-MB-231, MCF-7, K562 and A549 tumor cell lines. Amongst them, especially CA-4E showed the highest activity with IC_50_ values in the range of 1 to 180 nM. Activity of this novel compound was found to be more powerful than that of CA-4. Cell cycle results exhibited that CA-4E exert its effect in a time- and dose-dependent manner [82].

### 3.7. Effects on Tumor Histology of Combretastatins

Chemotherapeutic agents which have effects on the tumor microvasculature are remarkable at reducing tumor growth. This type of agents are called as vascular targeting or antivascular agents. Differently from antiangiogenic drugs, vascular targeting drugs affect particularly pre-existing tumor blood vessels. However, inhibition of proliferation of new blood vasculature of tumors is the main effect of antiangiogenic drugs. In contrast to antiangiogenesis strategy, antivascular agents can shut down of the established tumor vasculature rapidly and lead to secondary tumor cell death [83]. CA-4P is a prodrug that potently binds to tubulin microtubules of tumor microvessels specifically. CA-4P has been shown to give rise to increase in cellular necrosis in different tumor models and delay tumor growth. Single use of CA-4P as a vascular disrupting agent in several cancers such as ovarian, pancreatic neuroendocrine tumors, non-small cell lung cancer and glioma is well tolerated and adverse effects during the treatment are not serious [34].

It has been shown that a single intraperitoneal dose (100 mg/kg) of CA-4P to the mouse model of colorectal liver metastases decreased the density of patent vessels after 1 h from treatment and up to 24 h after one dose CA-4P. Loss of these vessels created a vascularity zone around the tumor. A single dose of CA-4P caused the heterogeneity of tumor areas due to absence of blood supply and microvessels. Regarding this effect on tumor vessels, a single dose (100 mg/kg) of CA-4P reduced the tumor blood flow and increased tumor necrosis. According to the results of this study, CA-4P is thought to be an effective vascular targeting agent that can change the tumor microvasculature and induce tumor necrosis [83].

In the study of Dark et al., i.v. injection of 100 mg/kg CA-4P into murine adenocarcinoma NT tumor and human breast carcinoma MDA-MB-231 tumor-bearing mice showed selective vascular shut down and extensive necrosis. Histological studies indicated a significant hemorrhagic necrosis consistent with widespread antivascular effects. Moreover, the results of this study revealed that CA-4P showed selective inhibition of tumor vasculature at doses less than one-tenth of the maximum tolerated dose thus demonstrating a wide therapeutic index [41].

Rapid reduction of tumor blood flow with the decrease of red cell velocity following the treatment of CA-4P was observed in the study performed by Tozer et al. In this study, microvascular effects of CA-4P have been investigated in the rat P22 tumor growing in a dorsal skin flap window chamber implanted into BD9 rats. Clumping of red blood cells to form rouleaux, abnormal rheology, and slowed blood flow were the indications of a vascular shut-down effect of CA-4P. Hemorrhage from peripheral vessels was also observed. Hemorrhage and fluid loss from the blood revealed that increase in tumor vascular permeability to macromolecules is an early effect of CA-4P. These findings reveal that combination of morphological and functional changes in endothelial cells cause the rapid decrease in tumor blood flow after the treatment of CA-4P in vivo. Rapid cell signaling between the tubulin and actin cytoskeletons leads to this effect of CA-4P [54].

CA-4P nanodrugs (CA-4-NPs) were developed which exerted more vascular disrupting effect than CA-4P for strong tumor growth inhibition. CA-4-NPs can accumulate around tumor blood vessels and shut down tumor microvasculature but it also significantly enhanced CXCR4 expression. The CXCR4/CXCL12 axis plays a crucial role in cancer metastasis. Malignant breast cancer cells can express the chemokine receptor CXCR4 and commonly metastasize to organs which contain large amount of CXCL12. Therefore, the blocking of the CXCR4/CXCL12 axis is an important approach in order to inhibit cancer metastasis in therapy. In the study of Jiang et al., 4T1 mammary adenocarcinoma cells injected female BALB/c mice were used for the investigation of effect of CA-4-NPs on tumor growth and metastasis simultaneously. The aim was to selectively shut down the formation of new blood vessels in tumor tissues using CA-4-NPs. Treatment of CA-4-NPs to the tumor tissue in mice increased the CXCR4 expression due to hypoxic conditions caused by CA-4-NPs. As a result of increased CXCR4 expression, the metastasis of tumor cells is also increased. To overcome this problem, CXCR4 antagonist, plerixafor, is combined with CA-4-NPs for tumor growth and metastasis inhibition simultaneously. The combination of CA-4-NPs + plerixafor exerted a significant tumor growth and metastasis inhibition compared with monotherapies. At this point, plerixafor showed strong inhibition of tumor cell metastasis due to disrupt the CXCR4/CXCL12 axis. Furthermore, in this study, it has been also exhibited the strong antitumor effect of CA-4-NPs + plerixafor combination without significant systematic cytotoxicity on normal organs according to the histological analysis [84].

Tang et al. revealed that CA-4P intensifies and worsens the symptoms of dextran sulfate sodium-induced ulcerative colitis in mice. As a result of this study it has been shown that CA-4P significantly reduced body weight and increased the disease activity index (DAI) of ulcerative colitis in mice. Aggravation of histological damage and inflammatory reactions was observed in the treatment of CA-4P in mice with ulcerative colitis. However, CA-4P also promoted inflammatory cell (neutrophil, monocyte, lymphocyte) levels and release of pro-inflammatory cytokines (IL-1β, IL-6, TNF-α) in dextran sulfate sodium-treated mice. On the other hand, resveratrol, a 1,2-diphenylethene derivative, can significantly suppress colitis and colon cancer associated with colitis but its bioavailability is poor due to low oral absorption and metabolism. Depending on this data, use of CA-4P on colitis treatment instead of resveratrol has been investigated by Tang et al. in this study. The study showed that despite both CA-4P and resveratrol belong to stilbenes and have similar structures, their activity on colitis treatment is not similar. Different biological effects of these compounds in colitis can be attributed to *cis-trans* isomerization of the compounds. CA-4P is a cis-stilbene while resveratrol is a *trans*-stilbene. According to the results, use of CA-4P for human cancer treatment is effective but the presence of ulcerative colitis or the risk of developing ulcerative colitis should be considered [34].

## 4. Nanoformulations of Combretastatins

Encapsulation technology is a process for entrapment of active molecules, drugs and agents which has been used for targeting, improving low water solubility, preserving stability and enhancing effectiveness of active compounds that are encapsulated in drug delivery systems such as nanoparticles, liposomes, polymer-drug conjugates, micelles, nanospheres, nanoemulsion, nanogels, dendrimers etc. which are the most widely prepared with polymers and lipids approved by Food and Drug Administration (FDA) [85,86].

Sengupta et al. designed new delivery system as named “nanocell” that contained nanoscale pegylated-phospholipid-poly-(lactic-co-glycolic) acid (PLGA) hybrid nanoparticles in order to target tumor cells and neovasculature at the same time. This system has a biodegradable and non-bioactive copolymer PLGA nanoparticles-conjugated anticancer drug doxorubicin and a lipid shell containing an antiangiogenic compound CA-4. The synergistic effect of these two drug was explained by assuming that firstly combretastatin was released for inhibition of the growth of tumor blood vessels and then the sustained release of cytotoxic agent doxorubicin from nanocells killed the tumor cells (B16/F10 melanoma and Lewis lung carcinoma) directly. When “nanocells” were used in a murine tumor model, CA-4 was first released to inhibit the growth of tumor blood vessels. The breakdown of the doxorubicin-PLGA conjugate provided sustained release of doxorubicin to kill tumor cells [87].

In another study, combination of CA-4 and doxorubicin was used for new polymeric micelle (PM) system development based on a ligand of integrins, cyclic arginine-glycine-aspartic acid-tyrosine-lysine pentapeptide (cRGDyK) was conjugated to the polyethylene glycol (PEG)-b-poly lactic acid (PEG-b-PLA) copolymers physically encapsulated with the antivascular agent CA-4 and chemically linked with the cytotoxic agent doxorubicin for treatment of tumor neovasculature. The targeted dual-drug micelle system containing CA-4, was increased cellular uptake of the drug by B16-F10 cells and endothelial cells of the human umbilical vein through a receptor-mediated endocytosis significantly. The double drug system modified with CRGDyK was obtained optimal antitumor effect, increased lifespan, induced antineovasculature, antiproliferation and apoptosis and represented the advantage of the modified combination therapy with the low dose of cytotoxic drug and targeted delivery systems [88].

Zhu et al. designed biodegradable polymersomes for co-entrappment of an antiangiogenic drug CA-4 and doxorubicin to destroy the tumor neovasculature and to inhibit cancer cell proliferation in order to achieve synergistic antitumor effects. The polymersomes were prepared by solvent evaporation method using methoxy poly(ethylene glycol)-b-polylactide (mPEG-PLA) block copolymers as drug carriers. Uniform vesicles around 50 nm in size were obtained and the best synergistic cytotoxic effect was obtained with a 1:10 doxorubicin:CA-4 ratio in a human nasopharyngeal epidermal carcinoma cell line. Also, polymersomes co-encapsulating doxorubicin and CA-4 (1:10) remarkably accumulated in human nasopharyngeal epidermal carcinoma tissues xenografts in nude mice and achieved antitumor effect significantly because of rapid tumor vasculature disruption and inhibition of sustained tumor cells proliferation. Consequently, their findings indicated that combination of an antiangiogenic drug and a chemotherapeutic agent in polymersomes is a potentially promising strategy for cancer treatment [89].

In another study involving a combination of drugs which were antiangiogenic agent (CA-4) and a chemotherapeutic drug (doxorubicin) co-loaded in a novel tumor vascular-targeting multidrug delivery system using mesoporous silica nanoparticles conjugated with targeting molecules (iRGD peptide) for anti-angiogenesis and chemotherapy. As an effective anti-angiogenesis agent CA-4 was used in this study because it is known to prevent tumor growth, spread, and disrupt tumor vasculatures. Also, due to the fact traditional chemotherapy leads to serious side effects and drug resistance, antiangiogenic agents are used as adjuvants. Significant releases at different rates of the antiangiogenic agent at tumor vasculature and the chemotherapeutic drug within the tumor cells were observed. It was observed that tumor growth was completely suppressed at an extremely low doxorubicin dose of 1.5 mg/kg in about 3 weeks with in vivo studies. A synergistic effect was demonstrated by the fast release of the antiangiogenic agent that caused disruption of the vascular structure, and after the slow release of the chemotherapeutic drug in the tumor area. Therefore, they said that existing drug delivery systems with double drug co-administration and tumor vascular targeting effect would be of great potential in the clinical treatment of cancer in the future [90].

An injectable nanogel-embedded hydrogel for synergistic therapy based on sequential local delivery of CA-4P and doxorubicin (DOX) was prepared by Yang and coworkers. Because of nanohydrogel’s hydrophilicity, flexibility, versatility, biocompatibility, morphology, and surface properties, kinetic release profile and sustained long-term release of drugs of nanometer-sized hydrogels have been utilized as drug carriers. In this study, firstly, the pH and redox stimuli-responsive poly (acrylic acid-co-4-vinylphenylboronic acid) nanohydrogels (P (AA-co-4-VPBA) NGs) were prepared with reflux-precipitation polymerization method. At the same time, the hydrophilic agent, CA-4P loaded the injectable hydrogel was designed for quick release. Then, for sustained long-term DOX delivery, DOX-loaded NG were incorporated into the injectable hydrogels through reversible chemical bonds (boronate esters) to fabricate a dual-drug delivery system (DOX-CA-4P@NHG). In vitro release study, CA-4P and DOX had different release profiles with release times of 80 h and 14 days, respectively. Controlled sequential release profiles were obtained with their purpose of developed nanohydrogels. MCF-7 cells and normal 3T3-L1 cell lines were used at cytotoxicity and cellular uptake studies. MCF-7 cells treated by dual drug delivery displayed higher DOX fluorescence intensities and efficient cellular uptake comparison with control group. Cancer cell proliferation was inhibited and combination therapy induced the highest apoptosis/necrosis of the cells in vitro. After a single injection of DOX-CA-4P@NHG, tumor samples were collected from the mice, an immunohistochemical analyses showed a synergistic therapeutic effect was achieved in that the antiangiogenic drug CA-4P was released quickly from the hydrogel, leading to the vascular disruption in tumor areas and slow release of cytotoxic DOX induced tumor cells apoptosis. The combination therapy of antiangiogenic and cytotoxic drugs coloaded- nanohydrogel delivery system was improved cancer therapeutic efficacy which can be used for dual-drug delivery with sequential release [91].

Numerous studies have reported that combinatorial therapy of targeted tumor cells is an effective approach and can use the synergistic effects of anti-angiogenesis and antitumor agents. Wang et al. fabricated a nanocapsule for delivery of CA-4 and paclitaxel (PTX) as vasculature targeting agent and conventional chemotherapeutic drug, respectively. They used methoxypoly (ethylene glycol)-poly (lactic acid) (mPEG-PLA) as the building-up matrix. The PTX was conjugated with ending group of PLA with cleavable ester bonds, and CA-4 was encapsulated in the core of the nanocapsules. Highly uniform and much smaller size nanocapsules approximately 70 nm in size were obtained and effective drug loading and sequential release of CA-4 and PTX were achieved with these nanocapsules. In vitro and in vivo studies were performed showing the significant therapeutic effects to malignant tumors. Enhanced cellular uptake and higher biodistribution with longer circulation of nanocapsule were seen in body fluids and hence, accumulation in the tumor area of nanocapsules was increased. Disruption of tumor vasculature, inhibition of tumor cell proliferation, and induction of tumor cell apoptosis were shown with both in vivo artificial proangiogenesis and tumor xenograft assays. The findings indicated that these nanocapsules containing CA-4 and PTX have potential in combinatorial cancer therapy and diagnosis [92].

Novel targeted CA-4-loaded micelles were prepared by Wang et al. from poly(ethylene glycol)-b-poly (d, l-lactide) (PEG-PLA) copolymers. RGD peptides that target integrins αvβ3 and αvβ5 that are markers of angiogenic endothelial cells, were coupled to the surface of micelles. The micelles were characterized in vitro and cellular uptake of micelles was evaluated by fluorometric determination and confocal microscopy. The SRB method was used for evaluation of anti-proliferation of targeted micelles. It was found that the mean diameters were 25.9 ± 1.3 nm, spherical micelles and entrapment efficiency of 97.2 ± 1.4%. In vitro release studies shown that sustained release from targeted micelles release occurred within 48 h. According to in vitro cellular uptake studies, significantly improved intracellular delivery of the CA-4 and increased anti-proliferation activity were obtained in angiogenic tumor endothelial cells via integrin-mediated endocytosis. Thus, RGD-conjugated CA-4-loaded PEG-PLA micelles have potential as a new formulation for targeting angiogenic tumor vasculature [93].

The vascular disrupting agent CA-4P has low oral absorption and nanoparticles are an approach to improve the oral absorption therefore in current study, Shen et al. were developed a novel nanoparticle formulation with methoxy poly(ethylene glycol)-b-polylactide (PELA) and poly(d,l-lactic-co-glycolic acid) (PLGA) polymers containing CA-4P for oral administration as an alternative to injectable forms. MDCK cell model which has been widely used in oral permeability studies was selected for in vitro transport study and in vivo antitumor effect was evaluated on S180 subcutaneous xenotransplanted tumor models. They found that novel CA-4P nanoparticles had better transcellular transport than free CA-4P, confirmed via permeability coefficient (Papp) values. CA-4P nanoparticles achieved an absolute bioavailability of 77.6% with the tumor inhibition ratio of 41.2% that was thought to significantly different with free CA-4P. Consequently, this work proved that biodegradable polymer-based nanoparticles are suitable carrier systems for small water-soluble compounds such as combretastatin and promising in oral administration [94]. Due to the poor permeability of nanoparticular systems in solid tumors, especially multiple drugs-loaded nanoparticles in the tumor region, new developments are needed for this.

Liu et al. improved novel nanoparticles for enhancing the tumor selectivity using PEGylated poly(α-lipoic acid) graft CA-4 (PALA-g-mPEG/CA-4) nanoparticle with glutathione (GSH) stimulus responsive ability was prepared from α-lipoic acid nanoparticles. CA-4 was linked to PALA-g-mPEG to obtain long blood circulation time and high GSH stimulus sensitivity. With this system, selective accumulation and release of CA-4 can occur in the tumor area. Cytotoxicity of free CA-4 and novel PEGylated poly(α-lipoic acid) graft CA-4 nanoparticles was measured with MTT test through 4T1 breast cancer cell line. The data suggested that PALA-g-mPEG/CA-4 provided the decreasing cytotoxic effect of free CA-4 and high anti-tumor activity to cancer cells with GSH sensitive release [95].

Lipid-based nanoemulsions are colloidal systems consisting of oil, surfactant and water and different therapeutic agents can be encapsulated in nanoemulsions. Mico and coworkers reported that encapsulation and in vitro delivery of CA-4 in lipid-stabilized oil nanodroplets (LONDs) which were prepared using seven different oils and their characterization parameters were evaluated. Optimum formulation components such as pressure, oil type were determined. Squalane and tripropionin were chosen as model oils because of created successful LONDs. Stability studies shown that squalane and tripropionin LONDs were stable for at least six weeks when kept at 4 °C, and for > 2 h at 37 °C. Successful drug release and intracellular uptake of CA-4 from LONDs were obtained and in resulting may be a promising step to effective treatments with CA-4 in vivo [96].

Liposomal formulations are a useful alternative to conventional drugs and very suitable lipid-based systems for targeted delivery in the treatment of many diseases. In many studies, the effectiveness of liposomes developed with CA-4 has been determined. In one of them, Nallamothu et al. prepared targeted liposomes using hydrogenated soybean (HSPC), cholesterol and distearoyl phosphoethanolamine-polyethylene-glycol-2000 (DSPE-PEG) conjugate and cyclic RGD (Arg-Gly-Asp) peptides were coupled to the distal end of PEG that formed long circulating liposomes. Liposomes were characterized and were examined using cultured human umbilical vein endothelial cells (HUVEC) in vitro. Encapsulation efficiencies (EE) % and particle size of liposomes were found to be 120 nm and 80%, respectively. Ligand coupling to the liposome surface was found to be more than 99% and targeted liposomes were bonded significantly more strongly to their target cells than nontargeted liposomes which improved the therapeutic effectiveness of CA-4 in treatment [97].

Jiang et al. developed a combination of curcumin and CA-4 liposomes to enhance the antitumor effect. They prepared glycyrrhetinic acid (GA)–modified and unmodified nanosized liposomes for liver-targeting and characterized them. Also, in vitro cellular uptake, cytotoxicity, cell migration, in vivo biodistribution, antitumor activity and histopathological studies were performed. Higher cytotoxicity than the free drugs with curcumin-CA-4/GA liposomes which were taken up effectively by human hepatocellular carcinoma cells (BEL-7402) was found. In vivo real-time near-infrared fluorescence–imaging results indicated increased GA-modified liposomes accumulation in the tumor region and inhibition of cell proliferation and an antitumor effect on hepatocellular carcinoma cells was exhibited [98].

In other some studies, liposome formulations containing CA-4 were prepared for different purposes such as immunoliposomes. Targeting anti-E-selectin conjugated immunoliposomes loaded with CA-4P to mice bearing transplanted MCa-4 mouse mammary tumors treated with therapeutic doses of ionizing radiation resulted in a significant delay in tumor growth when compared with other treatment groups such as free CA-4P, tumor irradiation alone, liposomal CA-4P alone and empty liposomes. Targeting of antivascular drugs to irradiated tumors via ligand-bearing liposomes results in significant tumor growth delay. Treatment combining fractionated radiation with fractionated CA-4P encapsulated immunoliposomes can be new clinical treatment approach [99].

Most of the liposomal drugs on the market or under clinical trials include cholesterol as a membrane stabilizing agent. Cholesterol content affects the physical and biochemical properties of liposomal membranes and indirectly the release of CA-4. In previous study, workers used liposomal formulations prepared with CA-4P, an antivascular drug, to evaluate the effect of cholesterol content on the release properties and cytotoxicity of CA-4 liposomes and according to their data, cholesterol was not only a membrane stabilizer, but it created a sterol superlattice form that affected the release and cytotoxicity of CA-4 from liposomes [100].

In another evaluation of a vasculature-targeted CA-4 liposomal formulation, liposome surface was modified with the hydrophilic and long polyethylene glycol (PEG) chains, called sterically stabilized liposomes (SSL) that could stay in circulation for a long time Ma et al. prepared CA-4-loaded vasculature-targeted sterically stabilized liposomes with Arg -Gly-Asp (RGD) peptide as a special ligand that provides targeting for the treatment of ocular angiogenesis like choroidal neovascularization (CNV), characterized in vitro, and evaluated the possibility of treatment of CNV in cell and animal models. Liposomes had an average diameter of ~ 120 nm, entrapment efficiency of over 70% and a relatively slow release. According to confocal images, a significantly enhanced cell uptake of liposomes by RGD modification and a decrease of CNV area were found. This suggests that the targeted liposome delivery system RGD-SSL-CA-4 can enhance the therapeutic efficacy of CA-4. Rats treated with 7 mg/kg RGD-SSL-CA-4, given for 2 weeks, resulted in laser-induced CNV that could be suppressed at a much lower dose. Because of targeting and long-term efficacy of the targeted liposome delivery system which is a new dosage form of CA-4 may be useful for the treatment of ocular angiogenesis diseases such as diabetic retinopathy and retinoblastoma as well as CNV [101].

Hao et al. reported that they developed pentapeptide Ala-Pro-Arg-Pro-Gly (APRPG)-PEG-poly(d,l-lactide) (PDLLA) and long-circulating agent monomethoxy poly(ethylene glycol) (MPEG) as pharmaceutic adjuvant that were approved by FDA, mixed micelles containing CA-4 to target tumor neovasculature for breast cancer therapy. Characterization parameters such as particle size, zeta potential, drug loading capacity, encapsulation efficiency, surface morphology, and crystallographic studies were investigated. An in vitro release study obtained a sustained release of CA-4 from the mixed micelles when compared to free CA-4. Also, the cytotoxicity data of blank and CA-4 loaded mixed micelles suggested that the APRPG-PEG-PDLLA/MPEG-PDLLA mixed micelles were safe drug carriers and the encapsulated CA-4 retained a potent antitumor effect. The cellular uptake study and the in vivo imaging and biodistribution study demonstrated that the APRPG peptide-modified mixed micelles have a higher cellular uptake efficiency and because of the active targeting property of the APRPG ligand, could significantly facilitate the drug accumulation in the tumor site. They concluded the CA-4 loaded APRPG-PEG-PDLLA/MPEG-PDLLA mixed micelles might be a new strategy for breast cancer therapy and could also be a potential carrier [102].

Shiraishi et al. aimed to evaluate tumor targeting of an antiangiogenic agent, a combretastatin derivative with adriamycin-encapsulated polymeric micelle carrier systems, containing either a diagnostic magnetic resonance imaging (MRI) contrast agent or a therapeutic anticancer drug. For tumor diagnosis by polymeric micelles, the MRI contrast agent can help estimation of the targeting efficiency of the polymeric micelle that encapsulates the anticancer-drug such as adriamycin-micelle. For the evaluation of effective therapeutic effect, the combination of a therapeutic drug and a diagnostic contrast agent included in the same carrier system called as “theragnosis” was used. Combretastatin derivative was applied 72 h before the application of the MRI agent to increase tumor targeting efficacy of the polymeric micelle carrier system. After this application, signal intensities in the tumor area was enhanced at 24 h after the MRI contrast agent injection. Combretastatin derivative pre-administration was increased tumor permeability and accumulation in tumor tissues and decreased tumor growth when using combination of adriamycin-encapsulating polymeric micelles in human scirrhous gastric carcinoma 44As3-bearing nude [103].

In another example, a CA-4 loaded-self-emulsifying drug delivery systems (SEDDS) was prepared and its performance evaluated. In preliminary studies, the appearance and particle size of emulsions with ternary phase diagrams and the solubility and dissolution characteristics of SEDDS was investigated in three different medias. The optimized formulation was determined to be CA-4-lauroglycol FCC-T20C-Transcutol P with a weight ratio of 3:50:35:15. The improved solubility and release of drug of CA-4 SEDDS were found to be 66 mg/mL^−1^ and 10 min, respectively. The SEDDS can clearly improve the solubility of CA-4 and the solubility properties of CA-4 SEDDS are not affected by different dissolution media [104].

Many advantages such as specific delivery of the drug to the targeting cells, regulated cytotoxicity, skipping drug resistance, reversing drug resistance, delivery of high drug loads are provided by attaching a small targeted ligand or peptide structure that binds to cytotoxic drugs for treatment of cancer. Bashari and coworkers focused metastatic castration-resistant prostate cancer (mCRPC) which is essentially incurable. The continual genetic evolution prostate cancer (PrC) cells are an additional complication that requires repeated patient monitoring to update chemotherapy regimens. Although it is an expensive process and drug toxicity, patient morbidity and survival are the issues to be considered. They aimed to treat mCRPC effectively with peptide-drug-conjugates (PDCs) conjugated with isolated PrC targeting peptides. PDCs were prepared with mono-drug (chlorambucil, combretastatin or camptothecin) and drug combinations (chlorambucil/ combretastatin or chlorambucil/camptothecin) and the cytotoxicity of these conjugates for target cells was tested with in vitro and in vivo studies. P12 peptide is active on a range of metastatic prostate cancer cell lines which specifically target PC-3 tumors, conjugated with drug combinations used for synergistic effect. It was concluded that using of these selected novel peptides as drug carriers for targeting drug delivery is possible. It is stated that PDCs may be an effective alternative to free drug chemotherapy for prostate cancer [105].

Polyamidoamine (PAMAM) dendrimers have uniform spherical shape with a suitable nanometer size, good water solubility, and biocompatibility, making them an ideal carrier system for drug delivery. In a recent study, for improving the water solubility and bioavailability, decreasing of toxicity of the CA-4, inclusion complexes were formed with dendrimer drug delivery system which has been demonstrated. They utilized generation 5 (G5) PAMAM dendrimers which is the first report of a new formulation for the CA-4, modified with fluorescein isothiocyanate and folic acid and with acetyl terminal groups (G5.NHAc-FI-FA). In conclusion, the inclusion complexes of G5.NHAc-FI-FA/CA-4 formed were able to improve the water solubility of the hydrophobic CA-4 significantly from 11.8 to 240 μg/mL, slow release rate of the drug was obtained and it said that inclusion complexes may act as a valuable carrier for targeting chemotherapy to different types of cancer and in vivo studies in the future [106].

## 5. Conclusions

Combretastatins, secondary metabolites in plants, are found to be beneficial in the treatment of several diseases. It is shown that these compounds have antimicrobial, antioxidant, anti-inflammatory, anticancer, and vascular properties. Combretastatin has emerged as fascinating scaffold for the development of new drugs. Many researchers are making continuous efforts for discovering combretastatin skeleton, especially for the improvement of anticancer activities. Taken together in vivo and in vitro studies, combretastatins have been shown to be promising candidates for the development of novel cancer therapeuticsr. Though several mechanism-based studies have been performed on their mechanism of action in cancer, this has yet to be fully explained. While several in vivo, and more recently, few clinical studies have evaluated the combretastatins biological effects, more efforts are needed to deepen knowledge in this field.
